# The Nonlinear and Gender-Related Relationships of Face Attractiveness and Typicality With Perceived Trustworthiness

**DOI:** 10.3389/fpsyg.2021.656084

**Published:** 2021-07-14

**Authors:** Nan Li, Ning Liu

**Affiliations:** ^1^School of Life Sciences, University of Science and Technology of China, Hefei, China; ^2^State Key Laboratory of Brain and Cognitive Science, Institute of Biophysics, Chinese Academy of Sciences (CAS), Beijing, China

**Keywords:** face perception, trustworthiness, attractiveness, typicality, gender differences

## Abstract

Perceived trustworthiness is one of the most important facial traits in social interaction. To elucidate how facial trustworthiness is assessed by others and its relationship to other facial traits would have significant theoretical and practical implications. Prior studies have shown that perceived attractiveness and typicality of a face may contribute to trustworthiness judgments; i.e., trustworthy faces are always the typical and attractive ones. Here, by conducting judgments of facial traits (i.e., trustworthiness, attractiveness, and typicality) on the same set of faces, we revealed a more profound relationship among these facial traits. First, we found that trustworthiness judgments did not always peak at the average face, in contrast to previous research. Second, trustworthiness exhibited a nonlinear relationship with attractiveness and typicality: Men relied more on typicality when judging a face as untrustworthy or neutral, whereas women relied more on typicality when judging a face as untrustworthy but more on attractiveness when judging a face as trustworthy. Third, women and men may utilize different traits to evaluate face trustworthiness: The relationship between trustworthiness and typicality judgments was closer in men than in women, whereas women counted on face attractiveness more than men did to evaluate face trustworthiness. These findings demonstrate that judging the trustworthiness of a face is a more complex process than previously thought, which may lead to a better understanding of the mechanisms underlying highly flexible and sophisticated social interactions in humans.

## Introduction

Faces convey a wealth of information (e.g., identity and personality traits) central to daily life. When meeting someone, “gut feelings” or first impressions are often rapidly and automatically formed based on facial appearance and then used to make inferences about personality traits, like trustworthiness and attractiveness (Zebrowitz, 2004; Todorov et al., 2005; van’t Wout and Sanfey, 2008; Antonakis and Dalgas, 2009). Such impressions influence a diverse range of critical social outcomes, from mate choice to sentencing decisions (Todorov et al., 2005). Therefore, it is important to understand what underlies face-based personality trait judgments.

Trustworthiness is important for judgments of a variety of personality traits. For instance, when multiple personality traits are represented in a 2D face evaluation space, judgments of trustworthiness account for over half of the variance in face-based social judgments and approximate the general evaluation of face valence (Oosterhof and Todorov, 2008). Prior studies have found that trustworthiness judgments exhibit high agreement among children and adults (Ma et al., 2016), indicating that certain common facial properties may play important roles in trustworthiness judgments. Identifying trustworthiness and its relationship to other face properties is critical for understanding the origin, evolution, and functional significance of trustworthiness.

Many studies have demonstrated that perceived attractiveness may contribute to trustworthiness judgments across races and cultures (Oosterhof and Todorov, 2008; Xu et al., 2012; Todorov et al., 2013). The “beauty is good” stereotype provides a possible explanation for this relationship (Langlois et al., 2000; Lemay et al., 2010): Attractive individuals represent health and fitness, as well as positive personality traits, such as honesty (Rhodes et al., 2007; Zebrowitz et al., 2011). Honesty is also an important component of trustworthiness (Langlois et al., 2000). Moreover, it has been found that facial textural properties (e.g., skin smoothness) directly affect physical attractiveness and indirectly affect trustworthiness (Tsankova and Kappas, 2016). In addition, for mate choice, physical attractiveness may trump judgments of personality traits, including trustworthiness (Fugère et al., 2017). Trustworthiness can be judged after as little as 50 ms of exposure to a neutral face (Todorov et al., 2009) and more quickly than many other personality traits (e.g., likeability, competence, and aggressiveness; Willis and Todorov, 2006), but not more quickly than attractiveness (Olson and Marshuetz, 2005). A recent study showed that attractiveness judgments precede trustworthiness judgments, with lower detection thresholds and shorter decision latencies (Gutierrez-Garcia et al., 2019). Moreover, prior research has found that the judgment-based event-related potential component occurs earlier for attractiveness than for trustworthiness (Calvo et al., 2018). Therefore, individuals may use attractiveness as an easily accessible proxy for judgments of trustworthiness. However, it remains unclear how and to what extent people employ attractiveness cues when judging trustworthiness from faces.

Trustworthiness judgments can also be influenced by face typicality, an important determinant of face recognition (Todorov et al., 2015; Dotsch et al., 2017; Sofer et al., 2017). Face typicality originates from Valentine’s norm-based face space model (Valentine, 1991), where all faces are represented as vectors originating from a typical, or average, face. The further a face is from the typical face, the more distinctive it is and the more easily it can be recognized. Trustworthiness judgments have been found to decrease with distance of computer-generated (morphed) faces from the typical face (Sofer et al., 2015) and to peak around the typical face of one’s own race (Sofer et al., 2017). The same shape of relationship has also been identified between face typicality and attractiveness (Langlois and Roggman, 1990; Rhodes, 2006). However, it has also been shown that the relationship between typicality and attractiveness dissociates to some degree. For example, Perrett and his colleagues found that the average (typical) face composed of a set of female faces is less attractive than an average composed specifically of attractive faces from the same set (Perrett et al., 1994). That is, the average face is attractive but may not be optimally attractive. As trustworthiness judgments are highly correlated with attractiveness judgments, one may wonder whether careful experimental design would reveal such a dissociation in the relationship between face trustworthiness and typicality. Moreover, as both face attractiveness and typicality are important factors in face trustworthiness, it is important to value them in trustworthiness judgments.

Previous studies have shown gender differences in various social behaviors (Derks et al., 2014; Mattarozzi et al., 2015; Lemmers-Jansen et al., 2019). Yet, surprisingly little research has been conducted on gender differences in face-based trustworthiness judgments. One study found that women tend to judge trustworthy faces significantly more trustworthy than men do, although no such differences were found for untrustworthy or neutral faces (Mattarozzi et al., 2015). In addition, women appear to respond more accurately (more in line with experimenter-defined dimensions) than men in trustworthiness judgment tasks (Dzhelyova et al., 2012). These findings tend to indicate that women and men may make face trustworthiness judgments by utilizing different traits, which remain unclear.

In the present study, we explored how trustworthiness judgments rely on attractiveness and typicality cues and whether these relationships are affected by the gender of the rater. In detail, we hypothesized that (1) perceived trustworthiness might not always peak at the average face, like attractiveness; (2) perceived trustworthiness might exhibit a nonlinear relationship with attractiveness and typicality; and (3) there might be a gender-related influence on the above-mentioned relationship.

## Materials and Methods

### Participants

In total, 119 adults participated in the present study, including (1) 40 participants [19–27 years, mean (*M*) ± standard deviation (*SD*) = 22.61 ± 2.67 years] in the face trustworthiness judgment task; (2) 39 participants (19–26 years, *M* = 21.87 ± 2.02 years) in the face attractiveness judgment task; and (3) 40 participants (19–28 years, *M* = 22.28 ± 2.36 years) in the facial typicality judgment task. Details on the study participants are shown in Table 1. Participants reported no abnormal neurological history, had normal or corrected-to-normal vision, and were right-handed. All participants provided written informed consent prior to the experiment and were compensated for their participation. All procedures were approved by the Institutional Review Board (2017-IRB-001).

**Table 1:** Demographic details of the participants.

Trait	Women			Men		
	*N*	Age range (years)	*M* ± *SD* (years)	*N*	Age range (years)	*M* ± *SD* (years)
Trustworthiness	20	19–27	22.71 ± 2.87	20	19–27	22.50 ± 2.52
Attractiveness	20	19–25	21.00 ± 1.49	19	19–26	22.89 ± 2.14
Typicality	21	19–26	21.29 ± 2.12	19	19–28	23.37 ± 2.17

All participants completed one of three tasks on the same set of face images. Given this between-subjects design, the sample size was determined on the basis of the desired power (0.80), alpha level (0.05), effect size (0.33), and number of groups (three in the main analysis). Using G*Power 3.1 (Faul et al., 2007), the minimum required sample size was calculated as 90.

### Stimuli

**Figure 1:**
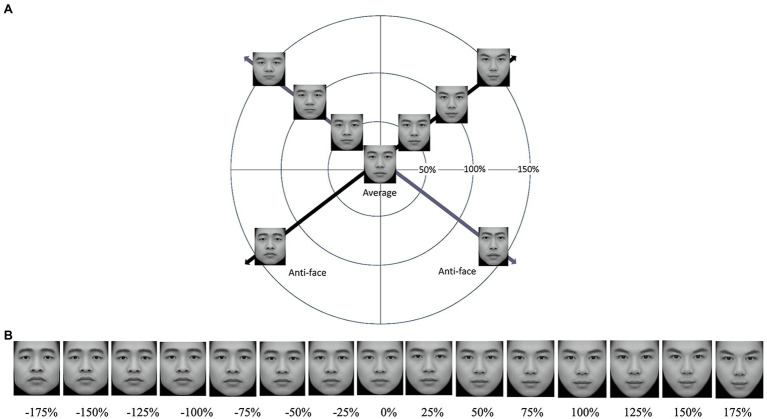
**(A)** Two face continua through the average face created by morphing 100 faces in a simplified face space. Anti-faces were made by morphing the original face toward and beyond the average face. **(B)** Example of one entire face continuum. Face transforms were created by adding or subtracting a percentage of the difference in shape between the average and original face. Thus, the average face was at the midpoint of the continuum (DFT = 0%), while the endpoints of the continuum were a caricatured face (DFT = +175%) and its anti-face (DFT = −175%).

Black-and-white photographs of young adults were selected from the CAS-PEAL-R1 Face Database (Gao et al., 2008) and SCUT-FBP5500 Database (Liang et al., 2018). Only faces from a frontal view and categorized as neutral faces were selected and utilized. Existing studies have found that women and men make similar trustworthiness and attractiveness judgments on male faces but not on female faces (Foos and Clark, 2011; Mattarozzi et al., 2015). Therefore, we used male faces only. One hundred faces were used to create an average (typical) face, with 20 of the hundred used in the main task and 15 in the practice. None of these faces were familiar to the participants. An average male composite (the typical face) was created using a standard procedure in Abrosoft FantaMorph v5 (Abrosoft Co., Beijing, China; Figure 1A). A set of landmark points (*n* = 194) was placed on each face to generate the average locations of each landmark. For faces used in the practice and main task, blemishes and hair on the forehead were removed using Adobe Photoshop (Adobe Systems, San Jose, CA, United States). The averaging process can make the composite face more symmetrical than the individual faces and thus affect judgments of facial traits. Therefore, to parse out the effect of averageness from the effect of symmetry, all individual faces were made perfectly symmetrical by averaging each face with its mirror image. All individual faces were standardized on the interocular distance of the average composite face.

For each face used in the main task, we constructed a continuum of faces (*n* = 14) by manipulating faces’ distance from the typical (average) face (Distance from the Typical face, DFT; Sofer et al., 2015) along individual dimensions from −175 to 175% in 25% steps (Figure 1B). The transformation between the corresponding landmark points (*n* = 194) of two faces is linear. All faces [*n* = 20 (original face) × 14 (level) = 280] had the texture of the average face. Each image was cropped tightly to the widest part of the face (thus removing the ears). All stimulus faces were presented against a uniform black background on a Dell P2217H, 22-inch monitor (1920 × 1080 pixels). When viewed from about 70 cm, images of faces subtended a visual angle of approximately 7.67° (H, ranging from 6.77° to 8.56°) × 6.62° (W, ranging from 5.62° to 7.61°).

### Procedure

**Figure 2:**
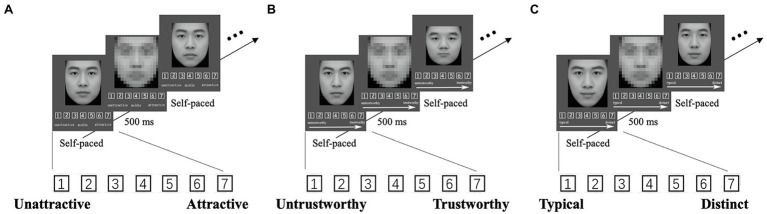
The procedure of face judgments. **(A)** The procedure of attractiveness judgments, **(B)** the procedure of trustworthiness judgments, and **(C)** the procedure of typicality judgments. A seven-point Likert scale was used for all trait judgments.

Experiments were conducted with MATLAB 2016a (MathWorks, Natick, MA) and PsychToolbox^1^ (Golarai et al., 2015). Participants were seated in a comfortable chair in a sound-attenuated room and randomly assigned to one of the three judgment tasks. To familiarize participants with their task, experiments started with a practice session, in which 15 faces were presented in random order. Participants were asked to rely on their “gut feeling” to rate faces on trustworthiness, attractiveness, or typicality using a seven-point Likert scale ranging from one (very untrustworthy/very unattractive/very typical) to seven (very trustworthy/very attractive/very distinct; Figure 2). They were encouraged to use the full range of the scale. Participants completed each trial at their own pace by clicking on the labeled buttons below each face. Once participants made the choice, a mask was presented for 500 ms to avoid the potential effect of visual adaptation on facial evaluation. In the main task, participants first previewed all 281 faces, which were presented for 800 ms each, followed by a mask for 500 ms. Then, after a three-minute break, they rated the 281 faces presented. To avoid participant fatigue, two rest intervals were implemented during testing. All faces were presented in a pseudo-randomized order (each particular face was constrained not to appear more than once per 20 trials; any particular level of faces constrained not to appear more than once per 14 trials) to eliminate the effect of familiarity on judgments of face traits.

## Results

### Agreement in Trait Judgments

Inter-rater agreement for the three kinds of trait judgments (i.e., trustworthiness, attractiveness, and typicality) was very high, with Cronbach’s alphas ranging from 0.98 to 0.99.

To further examine inter-rater agreement, we calculated Pearson’s correlations between each participant’s ratings and mean ratings across remaining individuals in their respective judgment groups (Kramer et al., 2018). Then, correlation coefficients were Fisher Z-transformed for normalization and tested against 0 with a one-sample *t*-test. We found that normalized correlation coefficients were significantly greater than zero (*p* < 0.001; Table 2), indicating that face-based trait judgments are highly reliable across participants. To explore the differences in judgment type and gender, we also performed a 3 (judgment type: trustworthiness, attractiveness, and typicality) × 2 (gender: women and men) two-way ANOVA followed up with *post-hoc* tests. The main effect of judgment type was significant [*F*(2,113) = 7.99, *p* < 0.001, partial *η*^2^ = 0.12]. *Post-hoc* (Bonferroni corrected) tests demonstrated no significant differences between agreements in attractiveness and typicality judgments, but both were significantly higher than that of trustworthiness judgments (compared with attractiveness: *p* = 0.002; compared with typicality: *p* = 0.003). These results suggest that trustworthiness may be a relatively complicated trait compared with attractiveness and typicality. We also found a significant main effect of gender [*F*(1,113) = 13.98, *p* < 0.001, partial *η*^2^ = 0.11], indicating higher agreement in judgments of facial traits among women than men. No interaction between judgment type and gender was found [*F*(2,113) = 0.05, *p* = 0.95, partial *η*^2^ = 0.001].

**Table 2:** Descriptive statistics on agreements (Z-value) in trait judgments (*M* ± *SD*).

	Trustworthiness	Attractiveness	Typicality
All	0.98 ± 0.24^∗∗∗^	1.14 ± 0.19^∗∗∗^	1.13 ± 0.19^∗∗∗^
Women	1.03 ± 0.24^∗∗∗^	1.21 ± 0.12^∗∗∗^	1.20 ± 0.16^∗∗∗^
Men	0.90 ± 0.23^∗∗∗^	1.05 ± 0.20^∗∗∗^	1.04 ± 0.19^∗∗∗^

∗∗∗ *p* < 0.001.

### Influence of DFT on Trait Judgments

We next explored the influence of DFT on participants’ judgments of trustworthiness, attractiveness, and typicality.

**Figure 3:**
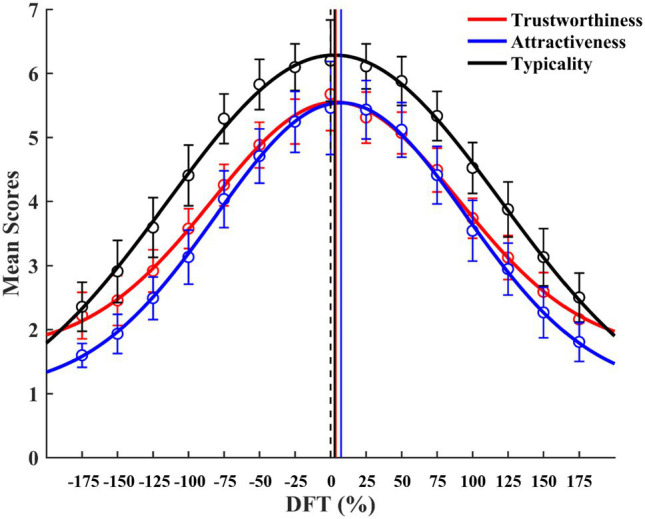
Mean trait judgments as a function of DFT based on all 20 tested face continua. Black vertical dotted line represents the location of the average face (DFT = 0%). Solid vertical lines represent locations of the predicted DFT for peaks of mean trustworthiness (red), attractiveness (blue), and typicality (black) judgments. Error bars represent 95% confidence intervals.

To identify the predicted DFT where facial trait judgments reached a maximum, Gaussian curves were fitted to mean judgments for all tested faces (Group All; DeBruine et al., 2007). For ease of presentation, ratings of face typicality were reversed so that the higher the rating, the more typical the face. For all three kinds of judgments, the Gaussian curve fits were very good, with *R*^2^ values of 0.93–0.95. Moreover, all three judgments peaked around the average face (trustworthiness: 3.64%; attractiveness: 7.41%; typicality: 2.80%; Figure 3). Thus, our results suggest that the average face is typical/trustworthy/attractive in general, as found in prior research (Langlois and Roggman, 1990; Todorov et al., 2015).

**Figure 4:**
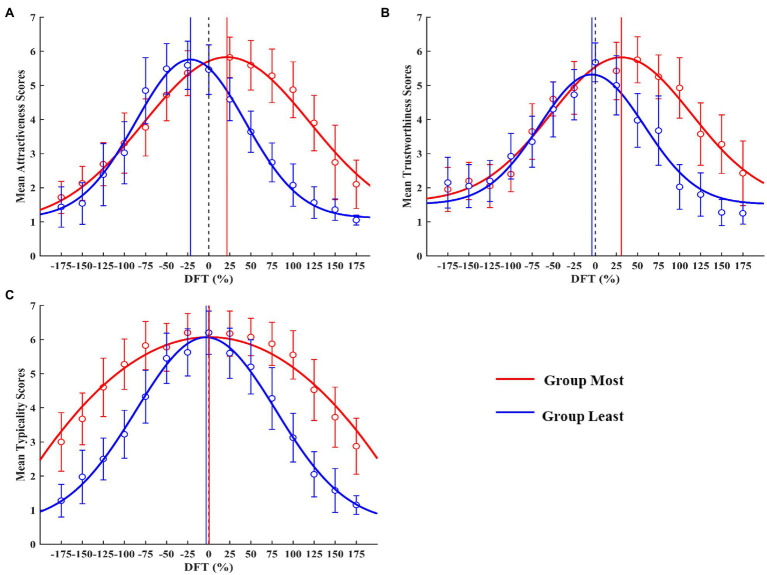
Mean trait judgments as a function of DFT based on Group Most/Least face continua: **(A)** attractiveness, **(B)** trustworthiness, and **(C)** typicality. Black dotted line represents the location of the average face (DFT = 0%). Solid lines represent locations of predicted DFT for peaks for Group Most (red) and Group Least (blue). Error bars represent 95% confidence intervals.

Previous studies have shown that attractive faces are not always average, whereas trustworthy faces are almost always average (DeBruine et al., 2007; Sofer et al., 2015). To re-examine these findings, we chose the most/least attractive original face based on mean participant ratings (most attractive rating = 4.87; least attractive rating = 2.08). Figure 4A shows the mean attractiveness judgments of the most/least attractive face continuum (called Group Most and Group Least, respectively) as a function of DFT. The predicted DFT for the peak of mean attractiveness judgments for Group Most and Group Least was distant from the average face (Group Most, peak = 21.64%; Group Least, peak = −21.54%). To confirm this impression, we fitted Gaussian curves to each participant’s data and calculated the predicted DFT for the peak of attractiveness judgment for each participant. A one-sample *t*-test against 0 was then conducted. Results showed that the predicted DFT for the peak of attractiveness judgments was indeed greater than 0% in Group Most (*p* < 0.001) and less than 0% in Group Least (*p* < 0.001). These results are consistent with previous findings that perceived attractiveness may not always peak at the average face (Sofer et al., 2015), confirming the reliability and effectiveness of the present method.

We then conducted similar analyses to examine whether trustworthiness would always peak around the average face. We chose the most/least trustworthy original face based on mean participant ratings (most trustworthy rating = 4.93; least trustworthy rating = 2.03). Figure 4B shows the mean trustworthiness judgments of the most/least trustworthy face continuum (called Group Most and Group Least, respectively) as a function of DFT. The predicted DFT for the peak of mean trustworthiness judgment for Group Most was distant from the average face (30.84%), whereas the peak for Group Least was close to the average face (−4.07%). Results of Gaussian curve fitting in each participant showed that the predicted DFT for the peak of trustworthiness judgments was indeed greater than 0% in Group Most (*p* < 0.001) but not different from 0% in Group Least (*p* = 0.456) indicating that, as attractiveness, perceived trustworthiness does not always peak at the average face.

To validate our method, we performed the same procedures for typicality judgments. We found that the predicted DFT for the peak of mean typicality judgments was indeed close to the average face in both Group Most (peak = 0.64%, *p* = 0.747) and Group Least (peak = 3.21%, *p* = 0.277; Figure 4C).

No significant differences were found when repeating the same analysis over data split based on the gender of the participants (Table 3).

**Table 3:** Descriptive statistics of the predicted DFT for peaks in trait judgments based on Gaussian curve fitting in each participant’s data (*M* ± *SD*).

Trait		All	Women	Men
Attractiveness	Group All	8.39 ± 14.31	4.66 ± 5.380	12.32 ± 10.88
	Group Most	23.24 ± 22.69	37.84 ± 15.38	28.83 ± 27.34
	Group Least	−21.28 ± 14.31	−22.78 ± 9.860	−19.70 ± 18.02
Trustworthiness	Group All	2.96 ± 10.36	3.56 ± 4.800	2.36 ± 14.02
	Group Most	20.99 ± 22.38	25.23 ± 14.59	16.75 ± 27.87
	Group Least	−2.19 ± 18.41	0.52 ± 20.92	−4.9 ± 15.58
Typicality	Group All	2.75 ± 7.270	1.24 ± 4.890	3.9 ± 9.070
	Group Most	−0.91 ± 16.89	1.33 ± 10.40	−1.7 ± 22.34
	Group Least	−1.96 ± 11.26	−3.93 ± 16.84	−0.96 ± 16.76

**Figure 5:**
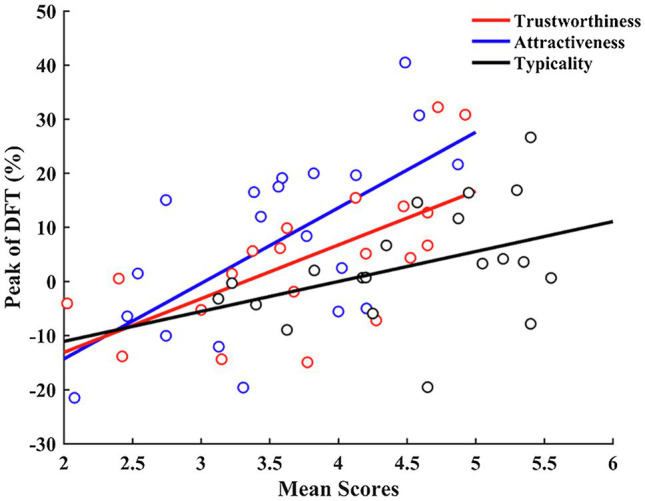
Relationships between mean trait ratings of original faces and the predicted DFT for peaks of mean trait judgments in corresponding continua. Lines represent best linear fits for trustworthiness (red), attractiveness (blue), and typicality (black) data.

As both the peak trustworthy and attractive faces are not always the average face, we wondered which trait was more related to face typicality. We fitted Gaussian curves to each original face continuum and calculated the corresponding predicted DFT for peaks of trustworthiness and attractiveness judgments. To model the relationship between mean trustworthiness/attractiveness ratings of original faces (Table 4) and the predicted DFT for peaks of mean trustworthiness/attractiveness judgments in corresponding continua, we fitted linear equations to the observed data (Figure 5). We conducted the same procedure for typicality judgments as a control. If one face-based trait judgment is mainly related to a face’s DFT, then for every face continuum this trait judgment should always peak at the average face and no significant correlation should be found between original faces’ ratings and the peaks of this trait judgment. Moreover, linear fitting slopes reflect the effect size of the DFT on trait judgments; i.e., the bigger the slope, the smaller the effect size.

**Table 4:** Descriptive statistics on trait judgments of each original face (*M* ± *SD*).

Attractiveness	Trustworthiness	Typicality
2.74 ± 1.14	3.23 ± 1.37	4.18 ± 1.38
4.49 ± 1.59	4.93 ± 1.38	5.40 ± 1.19
4.59 ± 1.83	3.63 ± 1.37	4.35 ± 1.42
3.82 ± 1.52	4.73 ± 1.41	4.95 ± 1.13
4.87 ± 1.26	4.53 ± 1.45	5.35 ± 1.23
3.59 ± 1.70	2.40 ± 1.24	3.23 ± 1.39
3.56 ± 1.50	3.58 ± 1.39	4.58 ± 1.34
2.08 ± 0.96	3.00 ± 1.26	3.40 ± 1.24
2.74 ± 1.70	2.03 ± 1.03	3.13 ± 1.11
4.03 ± 1.14	4.20 ± 1.47	5.20 ± 1.34
2.54 ± 1.21	2.43 ± 0.98	3.63 ± 1.31
3.77 ± 1.29	4.65 ± 1.27	5.05 ± 1.26
4.13 ± 1.59	4.48 ± 1.34	5.30 ± 1.14
4.00 ± 1.15	4.65 ± 1.41	5.55 ± 1.11
3.31 ± 1.06	3.78 ± 1.21	4.65 ± 1.29
3.13 ± 1.13	3.15 ± 1.33	4.25 ± 1.43
3.44 ± 1.47	3.68 ± 1.47	4.20 ± 1.30
4.21 ± 1.45	4.28 ± 1.34	5.40 ± 1.28
2.46 ± 1.10	3.38 ± 1.08	3.83 ± 1.34
3.38 ± 1.29	4.13 ± 1.30	4.88 ± 1.20

We did not find significant correlations for typicality judgments (*r* = 0.41, *p* = 0.075, *R*^2^ = 0.17, slope = 5.54); however, trustworthiness and attractiveness judgments exhibited significant correlations (for trustworthiness: *r* = 0.65, *p* = 0.002, *R*^2^ = 0.42, slope = 9.94; for attractiveness: *r* = 0.63, *p* = 0.003, *R*^2^ = 0.40, slope = 13.98). We utilized the bootstrap method (*n* = 1,000), resampling the participants to simulate the distributions of slopes if the experiments were repeated with different subjects. We found that the slope for trustworthiness judgments was significantly lower than that for attractiveness judgments (*p* = 0.037), suggesting that the relationship between trustworthiness and typicality may be closer than that between attractiveness and typicality.

To explore the effect of gender on relationships among trait judgments, we performed similar analyses for women and men separately. We found that the slope difference between attractiveness and trustworthiness judgments was significant in men but not in women (men, *p* = 0.029; women, *p* = 0.345; Table 5), indicating that there are gender differences in the role of typicality in face evaluation.

**Table 5:** Slopes of fitted lines for the relationship between mean trait ratings of original faces and the predicted DFT for peaks of mean trait judgments in corresponding continua by gender of the participants (*M* ± *SD*).

Trait	Women	Men
Attractiveness	11.15 ± 1.55	15.48 ± 3.18^*^
Trustworthiness	10.57 ± 1.15^###^	8.65 ± 1.39
Typicality	4.96 ± 0.99^^^^^	6.53 ± 1.59^^^^^

* *p* < 0.05 between attractiveness and trustworthiness judgments.

### *p* < 0.001 between trustworthiness and typicality judgments.

^^^ *p* < 0.001 between typicality and attractiveness judgments.

### Principal Component Analyses on Trait Judgments

**Figure 6:**
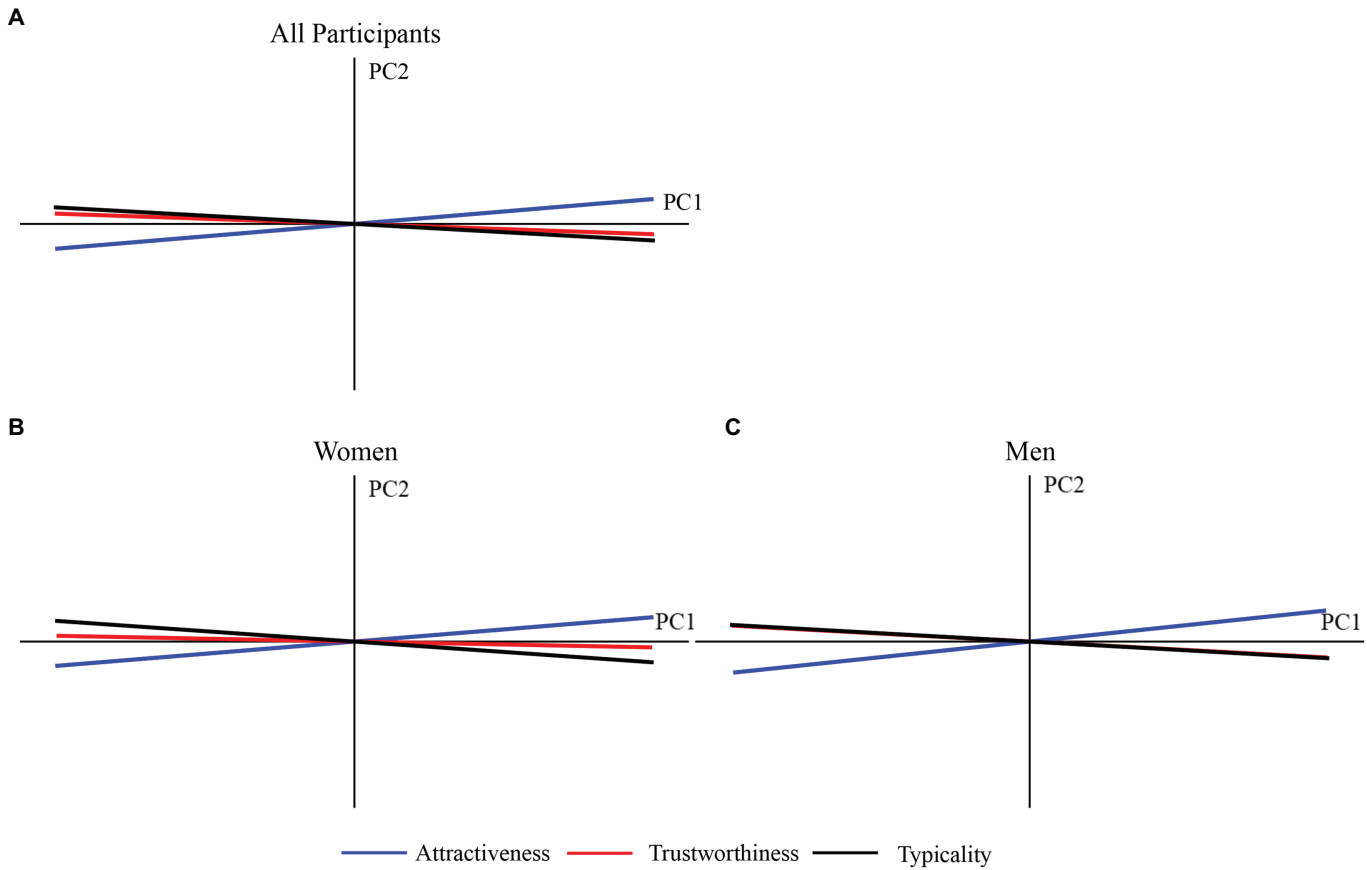
Principal component analyses of trait judgments across **(A)** all participants, **(B)** women only, and **(C)** men only. Space was built based on first two principal components (PCs; first PC: x-axis; second PC: y-axis). Locations of trait judgments are represented by their correlations with axes.

We also conducted principal component analyses (PCAs) on these trait judgments as described in the previous study (Oosterhof and Todorov, 2008), with mean ratings of the three trait judgments for all tested faces (*n* = 281). Based on the contribution to the variance of trait judgments, we choose the first two components. The first principal component (PC) accounted for 97.19% of variance, while the second PC accounted for 1.85% of variance. According to the previous study, the first component could be interpreted as the valence (trustworthiness) evaluation, while the second component could be interpreted as the dominance evaluation (Oosterhof and Todorov, 2008). For illustration purposes, we constructed a space based on the first two PCs with the locations of trait judgments represented by their correlations with these two PCs (Oosterhof and Todorov, 2008). In this space, the location of trustworthiness judgments was closer to that of typicality judgments than to that of attractiveness judgments (Figure 6A). These findings suggest that face typicality may be a more important determinant of perceived trustworthiness than attractiveness.

We conducted similar analyses for the two genders of participants separately. Principal component analyses showed that the relationship between perceived trustworthiness and typicality was closer in men than in women (Figures 6B,C). These results indicate that women may count more on perceived attractiveness than men to make facial trustworthiness judgments, whereas men might count more on perceived typicality than women to make trustworthiness judgments.

### Regression Models of Trustworthiness Judgments

**Figure 7:**
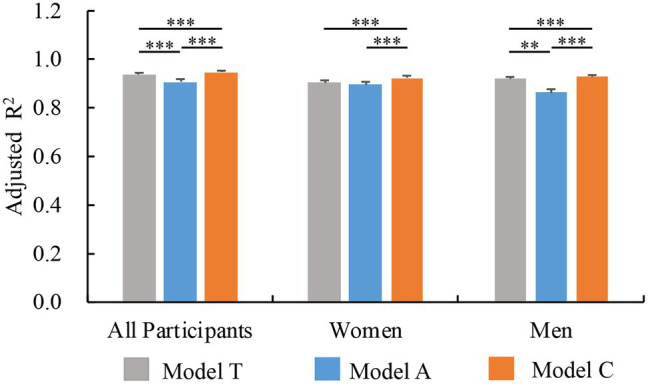
Performance of three regression models [typicality (Model T), attractiveness (Model A), and typicality and attractiveness combined (Model C)] of trustworthiness judgments based on all faces (*n* = 281) across all participants, women only, and men only. ^∗∗^*p <* 0.01; ^∗∗∗^*p* < 0.001. Error bars represent standard deviation.

To compare the roles of typicality and attractiveness in trustworthiness judgments, we conducted three regression models by implementing: (1) typicality (Model T); (2) attractiveness (Model A); and (3) typicality and attractiveness combined (Model C). We utilized the bootstrap method (*n* = 1,000), resampling the faces to simulate the distributions of performances (adjusted *R*^2^) of models if the experiments were repeated with different faces. We found that the performance of Model T was better than that of Model A (*p* < 0.001; Figure 7), indicating typicality may be more important for trustworthiness than attractiveness.

We conducted similar analyses for the two genders of participants separately and compared the models within and between genders. Using the bootstrap method (*n* = 1,000), we found that the performance of Model A was better in women than in men (*p* = 0.008), whereas the performance of Model T was better in men than in women (*p* = 0.033; Figure 7).

When analyzing influence of DFT on trait judgments, we found that the predicted DFT for the peak of trustworthiness judgments was not close to the average face in Group Most but was close in Group Least, whereas the predicted DFT for the peak of attractiveness judgments was not close to the average face in both Group Most and Group Least, indicating a potential nonlinear relationship between perceived trustworthiness and typicality/ attractiveness. To explore this possibility, we divided all tested faces into three sub-groups based on the mean ratings of face trustworthiness: Group Untrustworthy, faces with a mean score of < 3; Group Trustworthy, faces with a mean score of ≥ 5; and Group Neutral, all remaining faces. To exclude the potential effects of different numbers of faces across sub-groups on regression analyses, we randomly chose the same number of faces from each sub-group (*n* = 61) to perform the above regression analyses.

**Figure 8:**
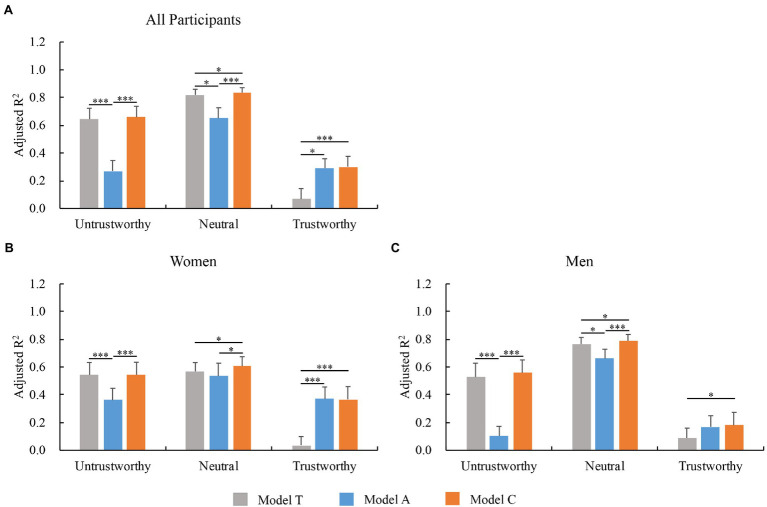
Performance of three regression models [typicality (Model T), attractiveness (Model A), and typicality and attractiveness combined (Model C)] for trustworthiness judgments across **(A)** all participants, **(B)** women only, and **(C)** men only based on faces with different trustworthiness ratings. All tested faces were divided into three sub-groups based on mean ratings of face trustworthiness: Group Untrustworthy, rating < 3; Group Neutral, 3 ≤ rating < 5; Group Trustworthy, rating ≥ 5. To exclude potential effects of different numbers of faces across sub-groups on regression analyses, we randomly chose the same number of faces (*n* = 61) from three sub-groups. ^∗^*p* < 0.05; ^∗∗∗^*p* < 0.001. Error bars represent standard deviation.

Figure 8A shows the performance of the three models for the different trustworthiness sub-groups. The statistical analysis method is the same as Figure 7. We utilized the bootstrap method (*n* = 1,000), resampling the faces to simulate the distributions of performances (adjusted *R*^2^) of Models if the experiments were repeated with different faces. We found that the performance of Model C in Group Trustworthy was significantly lower than the one in Group Untrustworthy (*p* = 0.002), and the latter one was significantly lower than the one in Group Neutral (*p =* 0.024), suggesting that people may need additional cues besides attractiveness and typicality to make non-neutral (untrustworthy and especially trustworthy) judgments of facial trustworthiness. The performance of Model T across three sub-groups showed the similar pattern as the one of Model C: Group Trustworthy vs. Group Untrustworthy, *p* < 0.001; Group Untrustworthy vs. Group Neutral, *p* = 0.059. However, the performance of Model A across three sub-groups showed a different pattern from those of Models C and T: The performance of Model A in Group Trustworthy was similar to the one in Group Untrustworthy (*p* = 0.479), and both of them were significantly lower than the one in Group Neutral (Group Untrustworthy: *p* = 0.002; Group Trustworthy: *p* < 0.001). Moreover, the performance of Model A was significantly lower than those for Model T in Group Untrustworthy and Neutral (Group Untrustworthy: *p* < 0.001; Group Neutral: *p* = 0.011). However, this phenomenon was reversed in Group Trustworthy (*p* = 0.015), indicating that people may rely more on attractiveness to judge trustworthy faces but rely more on typicality to make trustworthiness judgments of untrustworthy or neutral faces.

We next performed the above-mentioned analyses in women and men separately (Figures 8B,C). In Group Untrustworthy and Trustworthy but not in Group Neutral, adjusted *R*^2^ values of Model A were significantly or marginally higher in women than in men (Group Untrustworthy, *p* = 0.017; Group Trustworthy, *p* = 0.055). In Group Neutral but not in Group Untrustworthy and Trustworthy, the performance of Model T was significantly higher in men than in women (*p* = 0.005). These results indicate that women may count more on perceived attractiveness than men to trust or distrust faces, whereas men might count more on perceived typicality than women to make trustworthiness judgments of neutral faces.

## Discussion

In the present study, we investigated the relationship between trustworthiness and attractiveness/typicality judgments. Our results showed that trustworthy faces were not always typical. Moreover, we found that trustworthiness exhibited a complex nonlinear relationship with perceived attractiveness and typicality: Trustworthiness judgments relied more on attractiveness when judging trustworthy faces but relied more on typicality when judging neutral and untrustworthy faces. Furthermore, our study showed that there were gender differences in trustworthiness judgments. Below, we discuss the significance of these findings for understanding the underlying mechanisms of facial trait evaluation.

### Trustworthy Faces, Like Attractive Faces, Are Not Always Average

By determining the predicted DFT for peaks of trustworthiness judgments in different face continua (all faces, the most attractive face, and the most trustworthy face), we found that trustworthiness judgments in general (e.g., Group All) peaked around the average face. These findings are consistent with previous studies, which have reported that the relationship between face DFT and trustworthiness represents an inverted u-shape whenever natural faces, computer-generated faces, or a continuum of faces that vary on a typicality-attractiveness dimension are evaluated (Sofer et al., 2015, 2017; Todorov et al., 2015). However, it should be noted that in the most trustworthy face continuum (or the so-called typicality-trustworthiness dimension), which to the best of our knowledge has not been studied previously, trustworthiness judgments peaked away from the average face and toward the most trustworthy face. That is, an average face is trustworthy, but trustworthy faces are not always average. These results echo those found in the present study and previous research on judgments of attractiveness, which is a personality trait highly related to trustworthiness: Attractiveness judgments peak away from the average face and toward the most/least attractive face in the most/least attractive face continuum, respectively (DeBruine et al., 2007; Sofer et al., 2015). Our study compensates for the lack of trustworthiness judgments in the typicality-trustworthiness dimension and indicates that face typicality is important for trustworthiness judgments but to a smaller degree than previously thought (Sofer et al., 2015; Todorov et al., 2015).

Both trustworthiness and attractiveness judgments are important components of face evaluation. To further explore the role of face typicality in face evaluation, we compared values of face typicality for trustworthiness and attractiveness judgments. Our results showed that the trustworthiness/attractiveness ratings of each original face affected the predicted DFT of the trustworthiness/attractiveness judgment peaks in the corresponding face continuum, with a larger slope for attractiveness judgments than for trustworthiness ones. These findings indicate that the value of face typicality may be greater for trustworthiness judgments than for attractiveness judgments. These results are in line with locations of face trustworthiness and attractiveness in the 2D space of face evaluation defined by valence and dominance evaluation of faces, in which trustworthiness is closer to the valence dimension than attractiveness (Oosterhof and Todorov, 2008). Thus, the value of face typicality for one trait judgments may be associated with this trait judgment’s relationship with valence evaluation of faces: The closer the trait judgment to valence evaluation of faces, the larger the face typicality value. Our findings indicate a close link between face evaluation (especially valence evaluation) and face recognition, adding important information to the current body of knowledge regarding the role of typicality in face processing.

### Nonlinear Relationships of Face Attractiveness and Typicality With Perceived Trustworthiness

To explore and differentiate the roles of typicality and attractiveness in trustworthiness judgments, we constructed principal component analysis and multiple regression models. Our results showed that, in general, both face typicality and attractiveness predicted trustworthiness well, with better performance of typicality than attractiveness (Figures 6, 7). However, when we divided all tested faces into three sub-groups based on the mean ratings of face trustworthiness (Figure 8), we found that neither typicality nor attractiveness predicted trustworthiness when judging trustworthy faces as well as they did when judging neutral faces, indicating that additional information besides typicality and attractiveness is required in order to trust people based on faces. We also found that people relied on perceived attractiveness more than typicality when giving high trustworthiness ratings. Attractiveness may carry additional social information that cannot be explained by typicality, as reflected in the present work and prior studies that attractive faces are not always average (typical) faces (Valentine, 1991; DeBruine et al., 2007). Several facial traits have been proposed to influence both attractiveness and trustworthiness judgments, such as emotional expressions and feminine/masculine facial cues (Oosterhof and Todorov, 2008; Hu et al., 2018). These facial traits may contribute more to judge trustworthy faces than neutral faces. Though emotionally neutral faces were used in the present study, they may still show subtle cues of emotion (e.g., anger and happiness) that may contribute to perceived trustworthiness (Oosterhof and Todorov, 2008; Todorov and Engell, 2008; Lischke et al., 2018). Further studies are needed to explore the potential factors that are not clued by attractiveness and typicality but may affect judgments of trustworthy faces.

### Gender Differences in Trustworthiness Judgments

We also investigated potential differences in how people of different genders judge trustworthiness, which have been rarely studied. Our results showed that men relied more on typicality when judging a face as untrustworthy or neutral, whereas women relied more on typicality when judging a face as untrustworthy but more on attractiveness when judging a face as trustworthy. Moreover, we found that the relationship between trustworthiness and typicality judgments was closer in men than in women when judging neutral faces, whereas women counted on face attractiveness more than men did to evaluate face trustworthiness when judging trustworthy (and likely untrustworthy) faces.

Our data indicate that women and men may utilize different traits to evaluate face trustworthiness. Previous studies have shown the influence of gender on a broad range of social behaviors, including trust (Derks et al., 2014; Mattarozzi et al., 2015). It has been suggested that men are more trusting than women (Derks et al., 2014), though other research has cast doubt on gender differences in trust (Kanagaretnam et al., 2009). However, the role of gender of rater in facial trustworthiness judgments has been rarely studied. One previous study found that women are faster and more accurate in the perception of facial trustworthiness than men (Dzhelyova et al., 2012). Moreover, adaptation to facial trustworthiness occurs in women but not in men (Wincenciak et al., 2013). Here, we found that gender differences appeared as differential patterns in trustworthiness judgments under different judgment conditions. Since only male faces were used in the present study, we are unable to disentangle whether such gender differences are caused by different strategies of evaluating face trustworthiness between women and men or between same-gender and opposite-gender judgments. Moreover, previous studies have found that female faces are perceived to be more trustworthy than the male ones (Dong et al., 2018). Future studies could also incorporate female face stimuli and a cross-gender design to better understand gender effects on facial trait judgments.

Taken together, our study’s findings delineated the relationship between face trustworthiness and typicality/attractiveness in detail and found significant gender effects on trustworthiness judgments. Our findings demonstrated that to judge the trustworthiness of a face is a more complex process than previously thought, which may lead to a better understanding of the mechanisms underlying highly flexible and sophisticated social interactions in humans.

## Data Availability Statement

The raw data supporting the conclusions of this article will be made available by the authors, without undue reservation.

## Ethics Statement

The studies involving human participants were reviewed and approved by the Ethics Committee of the Institutional Review Board of Institute of Biophysics, Chinese Academy of Sciences (CAS). The patients/participants provided their written informed consent to participate in this study. Written informed consent was obtained from the individual(s) for the publication of any potentially identifiable images or data included in this article.

## Author Contributions

NLi and NLiu designed the experiments and drafted the manuscript. NLi performed the experiments and analyzed the data. NLiu supervised the data analysis. All the authors approved the final manuscript for submission.

### Conflict of Interest

The authors declare that the research was conducted in the absence of any commercial or financial relationships that could be construed as a potential conflict of interest.

## Footnotes

^1^http://psychtoolbox.org/
